# Modelling Optimal Control of Cholera in Communities Linked by Migration

**DOI:** 10.1155/2015/898264

**Published:** 2015-07-13

**Authors:** J. B. H. Njagarah, F. Nyabadza

**Affiliations:** Department of Mathematical Science, University of Stellenbosch, Private Bag X1, Matieland 7602, South Africa

## Abstract

A mathematical model for the dynamics of cholera transmission with permissible controls between two connected communities is developed and analysed. The dynamics of the disease in the adjacent communities are assumed to be similar, with the main differences only reflected in the transmission and disease related parameters. This assumption is based on the fact that adjacent communities often have different living conditions and movement is inclined toward the community with better living conditions. Community specific reproduction numbers are given assuming movement of those susceptible, infected, and recovered, between communities. We carry out sensitivity analysis of the model parameters using the Latin Hypercube Sampling scheme to ascertain the degree of effect the parameters and controls have on progression of the infection. Using principles from optimal control theory, a temporal relationship between the distribution of controls and severity of the infection is ascertained. Our results indicate that implementation of controls such as proper hygiene, sanitation, and vaccination across both affected communities is likely to annihilate the infection within half the time it would take through self-limitation. In addition, although an infection may still break out in the presence of controls, it may be up to 8 times less devastating when compared with the case when no controls are in place.

## 1. Introduction

Cholera is a gastroenteritic infection contracted after consuming the infectious dose or inoculum size of pathogenic* Vibrio cholerae*. The ability of* V. cholerae* to invade the gastroenteritis, proliferate, and damage the host characterises their virulence [1]. After invasion, the capacity of the vibrios to cause symptoms in the host characterises their pathogenicity. The transmission of vibrios from their natural habitat, the aquatic reservoirs, is through consumption of* Vibrio* infested water and seafood. This route forms the primary route of transmission. Cholera can also be transmitted from one person to another through consumption of unhygienic or soiled food that may be infested with pathogenic vibrios from an infected person. This route is characterised as the secondary route of transmission. For both of the mentioned routes, oral ingestion is the portal of entry of the pathogen into the human body.

Various controls for cholera have been recommended by the World Health Organization (WHO). These controls range from preventive measures to treatment protocols. Preventive measures are aimed at averting new infections by preventing the immunologically naive people from consuming or coming into contact with the bacteria. Treatment and control target the infected persons reducing the number of those infected as well as reducing the case fatality rate. Such preventive and control measures are explained in detail with regard to the metapopulation model of two connected communities as follows. First, an Oral Cholera Vaccine (OCV) recently recommended by the World Health Organization (WHO) [2] is now in use. Notably, it was recently used during the cholera outbreak that affected Haiti after the 2010-2011 earthquake [3]. Secondly, sanitation and hygiene reduce the rate of pathogen ingestion. Such controls include chemical treatment and boiling of water for cooking and drinking and proper storage and preparation of foods to prevent contamination. Thirdly, for the infected case management and treatment procedure which involves administering oral rehydration salts (ORS) to restore ion balance, intravenous administration of fluids in serious cases and use of antibiotics are recommended.

Mathematical modelling of cholera transmission dynamics dates back to the 1970s with the work done by Capasso and Paveri-Fontana [4], but more profound work has been done in the last 1 to 2 decades. Most of the work done so far focuses on specific communities and not the metapopulation framework of the infection. An extensive highlight of the metapopulation framework in epidemic models involving both cross community infections and exchange of populations between communities is given in [5] but not a single case is specific to cholera. Optimal control of the infection has only been studied recently in [6] when comparing cholera transmission between two separate Indian communities of Bogra and Calcutta. It is also important to note that optimal control of cholera in single community settings was studied in [6, 7]. To the best of our knowledge, no optimal control study has been considered in the metapopulation framework for cholera transmission to date. However, general modelling of control of epidemics in metapopulations was recently done using an SIS model [8]. In this same work, the authors highlighted the likely difficulty and mathematical intractability to be faced if a SIR or SIRS model is to be used. In this paper, we consider a SIRS model with controls since cholera transmission typically follows such dynamics. In addition a model of cholera transmission in a metapopulation setting published recently [9] highlights the detrimental effect of movement of epidemiologically naive and infected individuals across communities. In [9] however, the movement of recovered individuals was not considered. Given the length of the modelling time, we believe such movement is also possible and it is given consideration in this paper. We aim to consider the dynamics of closed and isolated communities in the presence of controls.

The paper is organised as follows. In Section 2, we present a metapopulation model with permissible controls. In the same section, vital mathematical analyses of the model are given including the community specific reproduction numbers followed by numerical results in Section 3 and in Section 5, we conclude the paper.

## 2. Mathematical Model

In the model, we consider two routes of transmission. The primary route is characterised by consumption of* Vibrio* infected water from aquatic sources. The secondary route (also referred to as person-to-person transmission) is characterised by consumption of food that may be contaminated with vibrios from faecal matter. The human population is subdivided into three compartments (*S*, *I*, and *R*) depending on their status with respect to the infection. The susceptible *S* are those who are at risk of contracting cholera either through contaminated water or by the secondary route. Once infected, individuals move into compartment *I*. Those who recover from the infection move into compartment *R* at rates *γ*_1_ and *γ*_2_ for the first and second compartments, respectively. Infected persons who show symptoms receive oral rehydration salts to restore the ion balance and this increases the recovery rate by a rate *r*. This increased recovery is assumed to be similar in both communities. The infection confers some temporary immunity which wanes at a rate *ω*. In the infection dynamics, the disease may be endemic or nonendemic. In the former case, the immunity of those infected wanes at a faster rate resulting in a SIRS type of model compared to a SIR model in the latter case. It is assumed that the time delay between consumption of* Vibrio* infected food or contaminated water and the commencement of infectiousness is negligible (see also [4]). The infectious dose or inoculum size *K* must be consumed if an immunologically naive person is to be infected. Given a high concentration of the infectious dose (*K* = 10^6^  cells  per  litre [10]) and the relatively low probability of infection, the dose-response relationship is given by the Monod function *β*_*i*_ *B*_*i*_/(*K* + *B*_*i*_) for *i* = {1,2}, where *K* (the inoculum size) is concentration of the pathogen required to cause 50% chance of infection. *β*_1_ and *β*_2_ are the specific contact rates of individuals in the first and second communities with the aquatic reservoirs in their corresponding communities. The use of a saturated incidence function *β*_*i*_ *B*_*i*_/(*K* + *B*_*i*_) for *K* > 0 ensures boundedness of the incidence rate and indicates that the increase in incidence rate is more gradual than linear. In the model we denote vaccination at any time *t* by *v*(*t*). We note that the immunologically naive individuals once vaccinated move straight to the compartment of those recovered. The likelihood of infection is reduced by fractions *u*(*t*) and *m*(*t*) related to contact with the aquatic reservoir and person-to-person contact, respectively. Here it is assumed that fraction *u*(*t*) uses clean treated water and fraction *m*(*t*) practises proper hygiene. We assume that the implementation of controls is by a government health organisation independent of the community specific health groups. Therefore, the implementation of controls may not be greatly influenced by the level of living condition in the adjacent communities but the need to contain the infection.

**Figure 1:**
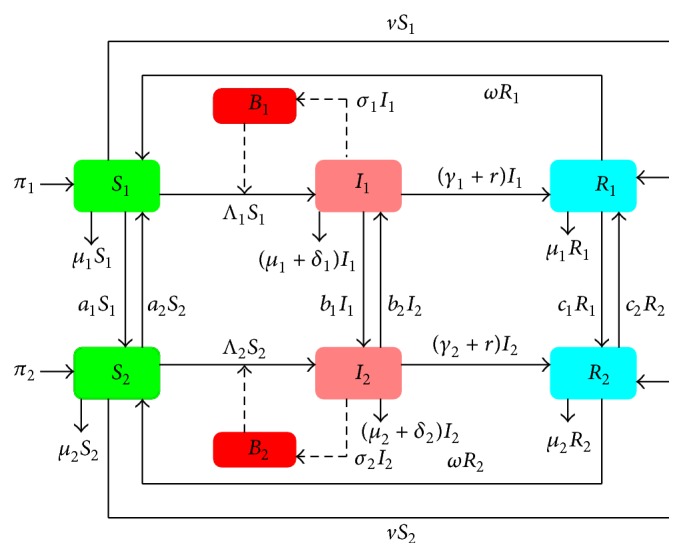
Flow diagram of disease dynamics in two communities.

The flow diagram of disease progression in two communities is given in Figure 1.

The terms Λ_1_ = (1 − *u*)(*β*_1_ *B*_1_/(*K* + *B*_1_)) + (1 − *m*)*α*_1_ *I*_1_ and Λ_2_ = (1 − *u*)(*β*_2_ *B*_2_/(*K* + *B*_2_)) + (1 − *m*)*α*_2_ *I*_2_ are the incidence functions for the first and second communities, respectively. These two terms describe the rate of apparition of new cholera cases in the respective communities.

The parameters *a*_1_, *b*_1_, and *c*_1_ are the respective rates of movement of susceptible, infected, and recovered individuals from the first community to the second community. Similarly *a*_2_, *b*_2_, and *c*_2_ are the rates of movement of the susceptible, infected, and recovered individuals from the second to the first community. The movement across communities is assumed to be instantaneous and therefore there is no change in epidemiological state during travel [5]. When either the susceptible, infected, or recovered individuals move from one community to the other, they follow the dynamics of the destination community. The secondary infections are only generated from within a community and there is no cross community infection.

The populations in the first and second communities are replenished through recruitments, at rates *π*_1_ and *π*_2_, respectively. Individuals in the two communities are, however, subjected to natural mortality at rates *μ*_1_ and *μ*_2_, respectively. The infected individuals in the two communities suffer disease induced mortality at rates *δ*_1_ and *δ*_2_, respectively. The pathogen concentration in the aquatic environment is replenished through shedding by the infected individuals at rates *σ*_1_ and *σ*_2_, for the two respective communities.

The model system of equations for the two communities in presence of controls is given by

(1a) dS1dt=π1+a2S2+ωR1−1−uβ1B1K+B1S1−1−mα1I1S1−a1+μ1+vS1,

(1b) dI1dt=1−uβ1B1K+B1S1+1−mα1I1S1+b2I2−Q1I1,

(1c) dR1dt=vS1+γ1+rI1+c2R2−μ1+ω+c1R1,

(1d) dB1dt=σ1I1−Q2B1,

(1e) dS2dt=π2+a1S1+ωR2−1−uβ2B2K+B2S2−1−mα2I2S2−a2+μ2+vS2,

(1f) dI2dt=1−uβ2B2K+B2S2+1−mα2I2S2+b1I1−Q3I2,

(1g) dR2dt=vS2+γ2+rI2+c1R1−μ2+ω+c2R2,

(1h) dB2dt=σ2I2−Q4B2,

where *Q*_1_ = (*μ*_1_ + *δ*_1_ + *γ*_1_ + *b*_1_ + *r*), *Q*_2_ = (*μ*_*p*_ − *g*_1_), *Q*_3_ = (*μ*_2_ + *δ*_2_ + *γ*_2_ + *b*_2_ + *r*), and *Q*_4_ = (*μ*_*p*_ − *g*_2_). Note that all constants in the balance equations are nonnegative. In addition *Q*_2_ and *Q*_4_ are positive, indicating that, in absence of faecal contribution from infected individuals, the bacteria cannot sustain themselves in the aquatic environment; see also [4]. The initial conditions of the model are *S*_10_, *I*_10_, *R*_10_, *B*_10_, *S*_20_, *I*_20_, *R*_20_, and *B*_20_ and are all nonnegative.

### 2.1. Model Analysis

The solutions to model system of (1a)–(1h) with nonnegative initial conditions are all nonnegative and bounded. Interested readers can investigate positivity and boundedness of the solutions using the method outlined in ([5, Chapter 3]). We note that if both communities are free of the infection, no treatment control protocol may be implemented. However, vaccination may still be in place as will be indicated in the steady states. Although the permissible controls vary with time, to analyse the steady states, we assume that the controls are constant thereby analysing a nonautonomous system of differential equations; see also [7]. Therefore, the disease-free equilibrium *𝔼*_0_ is given by

(2) $$\begin{array}{c} E_{0}=\left\{\;S_{1}^{*},0,0,0,S_{2}^{*},0,0,0\;\right\}, \end{array}$$

where

(3) S1=π1a2+μ2+v+a2π2a1+μ1+va2+μ2+v1−Φ1,S2=π2a1+μ1+v+a1π1a1+μ1+va2+μ2+v1−Φ1.

The term Φ_1_ = *a*_1_/(*a*_1_ + *μ*_1_ + *v*) · *a*_2_/(*a*_2_ + *μ*_2_ + *v*) depicts the proportion of susceptible individuals who move back and forth in compartments *S*_1_ and *S*_2_. Therefore, (1 − Φ_1_) is the fraction of susceptible individuals who do not move from their home compartments. We note also that the proportions of the susceptible *S*_1_ and *S*_2_ fall off as quadratic terms of the vaccination (in the denominator) and increase linearly (in the numerator). Therefore, the higher the vaccination coverage, the lower the fraction of the population that remains naive to the infection.

The community specific disease threshold numbers can be obtained using the next generation matrix method outlined in [20]. When computing the disease thresholds however, we assume that the controls are constant so that the model system of equations is nonautonomous:

(4) R01oc=π1a2+μ2+v+a2π21−mα1Q2K+1−uβ1σ1Q1Q2a1+μ1+va2+μ2+v1−Φ1K

for the first community and

(5) R02oc=π2a1+μ1+v+a1π11−mα2Q4K+1−uβ2σ2Q3Q4a1+μ1+va2+μ2+v1−Φ1K

for the second community. Then the model basic reproduction *ℛ*_0oc_ is given by

(6) $$\begin{array}{c} R_{0\text{oc}}=\max\left\{\;R_{01\text{oc}},R_{02\text{oc}}\;\right\}. \end{array}$$

**Theorem 1 .**

The disease-free steady state (2) of model of (1a)–(1h) is globally asymptotically stable whenever *ℛ*_0oc_ < 1 and unstable otherwise.

The proof of Theorem 1 can be given using the approach to the proof for Lemma  1 in [9]. However, in this case the community specific reproduction numbers to be used are (4) and (5).

### 2.2. Optimal Control

The general procedure of optimal control process in an epidemiological model involves the following processes: (1) identifying permissible controls applicable to the model, (2) setting up the objective function with controls, (3) constructing the Hamiltonian, (4) evaluating costate variable (adjoint functions), and (5) identifying the threshold controls that minimise the Hamiltonian. This optimal control minimisation procedure follows Pontryagin's Maximum/Minimum principle [21].

The objective function for our minimisation problem is given by

(7) $$\begin{array}{c} J\left(\;u,v,m\;\right)=\overset{T}{\underset{0}{\int }}\left(\;\xi_{1}I_{1}+\xi_{2}I_{2}+\chi u^{2}+yv^{2}+zm^{2}\;\right)dt. \end{array}$$

The coefficients *ξ*_1_, *ξ*_2_, *χ*, *y*, and *z* are the coefficients associated with the costs over a finite period of time *T*. *ξ*_1_ *I*_1_ and *ξ*_2_ *I*_2_ indicate the cost associated with minimising the infection in the first and second communities, respectively. *χ*, *y*, and *z* are relative cost weights for the respective control measures. We assume that the cost of controls is nonlinear, hence the use of the quadratic terms. The main goal is to minimise the number of those infected in both communities at the same time minimising the cost of implementing the controls. In this respect, we seek optimal controls *u* ^*∗*^, *v* ^*∗*^, and *m* ^*∗*^ such that

(8) $$\begin{array}{c} J\left(\;u^{*},v^{*},m^{*}\;\right)=\underset{u,v,m}{\min}\left\{\;J\left(\;u,v,m\;\right)\;|\;u,v,m\in U\;\right\}, \end{array}$$

where

(9) $$\begin{array}{c} U≔\left\{\;\left(\;u,v,m\;\right)\;|\;u,v,m:\left[\;0,T\;\right]⟶\left[\;0,1\;\right],\;\;\forall u,v,m\;\right\}. \end{array}$$

To evaluate the integral in (7), we use the Hamiltonian constructed for the model system of equations with the necessary adjoint functions. Let *λ*_*S*_1__, *λ*_*I*_1__, *λ*_*R*_1__, *λ*_*B*_1__, *λ*_*S*_2__, *λ*_*I*_2__, *λ*_*R*_2__, and *λ*_*B*_2__ be the adjoint functions or costate variables associated with the evolution of the corresponding state variables. We multiply each of the adjoint functions with the right side of the equation describing the evolution of each state variable:

(10) H=ξ1I1+ξ2I2+χu2+yv2+zm2+λS1π1+a2S2+ωR1−1−uβ1B1K+B1S1−1−mα1I1S1−a1+μ1+vS1+λI11−uβ1B1K+B1S1+1−mα1I1S1+b2I2−Q1I1+λR1vS1+γ1I1+rI1+c2R2−μ1+ω+c1R1+λB1σ1I1−Q2B1+λS2π2+a1S1+ωR2−1−uβ2B2K+B2S2−1−mα2I2S2−a2+μ2S2−vS2+λI21−uβ2B2K+B2S2+1−mα2I2S2+b1I1−Q3I2+λR2vS2+γ2I2+c1R1−μ2+ω+c2R2+λR2σ2I2−Q4B2.

**Theorem 2 (see [22]).**

Let *u* ^*∗*^, *v* ^*∗*^, and *m* ^*∗*^ be the optimal controls for system (1a)–(1h), *x* ^*∗*^ the state space at equilibrium, and *λ*_(•)_ positive semidefinite piecewise differentiable functions for all *t*. If one supposes that, for all *t* ∈ [0, *T*],

(11) 0Hut,x∗,u∗,v∗,m∗,λt=Hvt,x∗,u∗,v∗,m∗,λt=Hmt,x∗,u∗,v∗,m∗,λt

then

(12) Ht,x∗,u∗t,v∗t,m∗t,λt≤Ht,x∗,ut,vt,mt,λt

holds for the optimal controls *u* ^*∗*^, *v* ^*∗*^, and *m* ^*∗*^.

To find the differential equations with respect to the associated adjoint functions, we differentiate the Hamiltonian with respect to each of the state variables and obtain

(13) dλS1dt=1−uβ1B1K+B1+1−mα1I1λS1−λI1+a1+μ1+vλS1−vλR1−a1λS2,dλI1dt=1−mα1S1λS1−λI1−ξ1+Q1λI1−γ1+rλR1−σ1λB1−b1λI2,dλR1dt=μ1+ω+c1λR1−ωλS1−c2λR2,dλB1dt=1−uβ1S1KK+B12λS1−λI1+Q2λB1,dλS2dt=1−uβ1B2K+B2+1−mα2I2λS2−λI2+a2+μ2+vλS2−vλR2−a2λS1,dλI2dt=1−mα2S2λS2−λI2−ξ2+Q3λI2−γ2+rλR2−σ2λB2−b2λI1,dλR2dt=μ2+ω+c2λR2−ωλS2−c1λR1,dλB1dt=1−uβ2S2KK+B22λS2−λI2+Q4λB2,

with transversality condition

(14) λS1TλI1T=λR1T=λB1T=λS2T=λI2T=λR2T=λB2T=0.

We note that for given transversality condition (14) the following hold:

(15) $$\begin{array}{c} \frac{d\lambda_{S_{1}}}{dt}=-\frac{\partial H}{dS_{1}},\\ \vdots \\ \frac{d\lambda_{B_{2}}}{dt}=-\frac{\partial H}{dB_{2}}. \end{array}$$

The optimal controls are characterised by the following expressions:

(16) u∗t=max⁡0,min⁡u^t,1,v∗t=max⁡0,min⁡v^t,1,m∗t=max⁡0,min⁡m^t,1.

The standard control arguments on the controls [23] are such that

(17) $$\begin{array}{c} u^{*}=\left\{\begin{array}{cc} 0 & \text{if }\hat{u}\leq 0,\\ \hat{u} & \text{if }0<\hat{u}<1,\\ 1 & \text{if }\hat{u}\geq 1. \end{array}\right. \end{array}$$

The upper bound of *u* ^*∗*^ indicates that drinkable water is least likely to be responsible for the oral faecal route of transmitting the infection, especially if water is chlorinated, disinfected, or boiled:

(18) $$\begin{array}{c} v^{*}=\left\{\begin{array}{cc} 0 & \text{if }\hat{v}\leq 0,\\ \hat{v} & \text{if }0<\hat{v}<1,\\ 1 & \text{if }\hat{v}\geq 1. \end{array}\right. \end{array}$$

The value *v* ^*∗*^ = 1 is characteristic of a perfectly effective vaccine. Quite often cholera vaccines have low protective efficacy and have adverse effects associated with them. In one study by the Public Health Agency of Canada [24], the efficacy of the cholera and diarrhoeal vaccine was observed to range between 64% and 85% against* Vibrio cholerae* 01 El Tor. Therefore, the upper bound (*v* ^*∗*^ = 1) of this control may not be necessarily attainable:

(19) $$\begin{array}{c} m^{*}=\left\{\begin{array}{cc} 0 & \text{if }\hat{m}\leq 0,\\ \hat{m} & \text{if }0<\hat{m}<1,\\ 1 & \text{if }\hat{m}\geq 1. \end{array}\right. \end{array}$$

Similarly, *m* ^*∗*^ = 1 would signify no transmission of the pathogen through consumption of foods and that all foodstuffs are well prepared and hygienically stored and free from contamination. Unfortunately, this is not usually the case in communities where cholera is endemic.

Differentiating *ℋ* with respect to each of the admissible controls, we obtain

(20) ∂H∂u=2χu+λS2−λI2β2B2S2K+B2+λS1−λI1β1B1S1K+B1,∂H∂v=2yv+λR1−λS1S1+λR2−λS2S2,∂H∂m=2zm+λS1−λI1α1I1S1+λS2−λI2α2I2S2.

The control characterisations, $\hat{u}$, $\hat{v}$, and $\hat{m}$, of the optimal controls *u* ^*∗*^, *v* ^*∗*^, and *m* ^*∗*^ are obtained from ∂*ℋ*/∂*u* = ∂*ℋ*/∂*v* = ∂*ℋ*/∂*m* = 0:

(21) u^=λI2−λS2β2B2S2/K+B2+λI1−λS1β1B1S1/K+B12χ,v^=λS1−λR1S1+λS2−λR2S22y,m^=λI1−λS1α1I1S1+λI2−λS2α2I2S22z.

It can be noted that the control characterisations are inversely proportional to the associated costs. The costs are therefore influential in establishing the levels and trajectories of the controls.

The optimal values of the controls are the ones anticipated to contain the disease. This is also in accordance with the other model parameters describing the dynamics of the disease. The incidence of the disease is also key in determining the trajectory of the state variables for the model without controls, the model with controls, and the control variables over time.

## 3. Numerical Simulations

To numerically solve the optimal control problem, we modify a code developed by Lenhart and Workman [22], to accommodate the model system of (1a)–(1h). We note that this optimal control problem is classified as a quadratic programming problem, since the controls are nonlinear and are of a quadratic form. The state space variables are solved forward in time and the adjoints associated with the state variables solved backward in time. The model system of equations is numerically integrated using the ODE integration routine (ode45), a fourth-order Runge-Kutta Method in MATLAB.

### 3.1. Parameter Estimation

Due to lack of data, the parameter values used are estimated from published literature and some intuitively selected from information related to cholera transmission dynamics. The unit of the parameters is per day except for those where otherwise is indicated. Nominal values are selected from within the specified ranges to cater for the differences in the communities under study as well as the disease transmission dynamics. In the model, we assume that the first community has better living conditions and better facilities. Therefore, the transmission parameters are assumed to be relatively lower and the infection is assumed to be less devastating in the first community. The second community, however, is assumed to be slightly less disadvantaged and the infection more devastating. Because of the difference in assumed living conditions, migration of individuals is assumed to be inclined towards the first community. The parameters related to movement are set such that *a*_2_ > *a*_1_, *b*_2_ > *b*_1_, and *c*_2_ > *c*_1_. It is also necessary to determine the parameters related to the cost in the objective function (7). The estimated cost of a vaccine dose per person from the study in [19] was about $5. This gives an estimated cost of 50 rands within the South African context. We note that access to clean water and proper sanitation are influenced by a number of factors ranging from the cost associated, the standard of water treatment, and the sophistication of the technology used. According to the study conducted in Johannesburg [18], if all households were to access clean in-house piped water, with proper monitoring and in-house sewage connection with partial treatment, it would cost about US $136.5 billion per year. According to Statistics South Africa [25], the 2014 mid-year population estimate in the country is 54 million. This implies that the estimated cost of access to proper sanitation per person per day is approximately US $6.925. This is equivalent to approximately R80. In the case of infection, the average cost per percentage reduction in the infection in a community is influenced by factors such as the cost of oral rehydration salts, advertisement on social media, transportation, and remuneration. Given the difference of severity of the infection in adjacent communities, we assume that the cost is lower in a community with better facilities. We assume an average cost of R200 in a more devastated community compared to R120 in a community with improved facilities. The estimated costs associated with the controls per person are given in Table 2.

## 4. Sensitivity Analysis

We analyse the sensitivity of the model community specific reproduction numbers (*ℛ*_01oc_ and *ℛ*_02oc_) to the model parameters using the Latin Hypercube Sampling (LHS) scheme with 1000 runs per simulation. The LHS is a Monte Carlo stratified sampling method that allows us to simultaneously obtain an unbiased estimate of the model output for a given set of input parameter values; see [26]. This statistical sampling method for simultaneously carrying out sensitivity and uncertainty analysis was first applied in an epidemiological model by Blower and Dowlatabadi [27]. The approach has since been employed in a number of epidemiological models including [9, 26].

**Figure 2:**
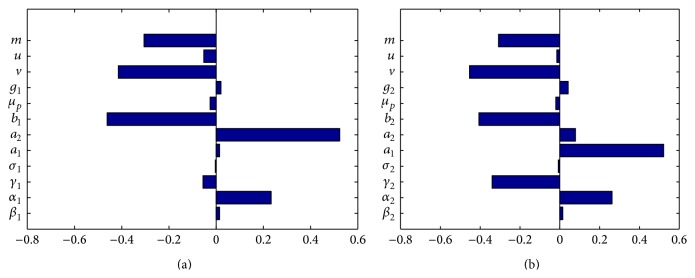
Partial Rank Correlation Coefficients (PRCCs) for the full range of parameters from Table 1. The control parameters with the greatest potential of containing the infection are improved hygiene and vaccination, while the influx of susceptible individual in either community increases the likelihood of the disease outbreak.

The Partial Rank Correlation Coefficients (PRCCs) for the full range of parameters are shown in the tornado plots, Figure 2(a) for the first community and Figure 2(b) for the second community. The PRCCs for the community specific reproduction numbers in Figures 2(a) and 2(b) are produced using the expressions *ℛ*_01oc_ and *ℛ*_02oc_. The process associated with parameters with positive (negative) PRCCs has the potential of aggravating (reducing) the infection when increased. From the equations of the community specific reproduction numbers *ℛ*_01oc_ and *ℛ*_02oc_, the controls parameters are evident. From the tornado plots in Figure 2 for community specific disease thresholds, it is evident that the controls related to increasing access to clean water and enhancing proper hand hygiene reducing the disease threshold are all associated with negative PRCCs. Therefore, with enhancement of processes related to such controls, the subsequent numbers of those infected will be less than that of their predecessors hence reducing the severity of the infection. It should be noted that, of all the considered controls, vaccination and improvement in hygiene have the greatest potential of easily containing the infection. In addition emigration (immigration) of infected individuals out of (into) a community reduces (increases) the disease burden in that community.

### 4.1. Numerical Results

In the model simulations, the population is not constant and the initial conditions are set as proportions as follows: *S*_10_ = 0.6; *I*_10_ = 0.0002; *R*_10_ = 0.002; *B*_10_ = 1 × 10^11^; *S*_20_ = 0.8; *I*_20_ = 0.003; *R*_20_ = 0.002; *B*_20_ = 1 × 10^11^.

The algorithm for simulating the system using the “forward-backward sweep method” is adopted from [22]. We outline the steps followed during the simulation for convenience of the reader.

**Step 1 .**

The time interval over which the system is to be integrated is subdivided into *N* equal subintervals. The initial guesses of piecewise-constant controls *u*_0_(*t*), *v*_0_(*t*), and *m*_0_(*t*) on the interval *t* ∈ [*t*_*i*_, *t*_*i*+1_], for *i* = 0,1,…, *N*, are assumed.

**Step 2 .**

Using the assumed controls *u*_0_, *v*_0_, and *m*_0_ and the initial conditions $\vec{x}(0)=\left(S_{10},I_{10},R_{10},B_{10},S_{20},I_{20},R_{20},B_{20}\right)$ of the model, the system is numerically integrated forward in time [0, *T*] according to its system of differential equations in the optimality system.

**Step 3 .**

Using the assumed controls *u*_0_(*t*), *v*_0_(*t*), and *m*_0_(*t*) and values of $\vec{x}$, the costate system $\vec{\lambda }(t)$ in accordance with its system of differential equations with the transversality condition is integrated backward in time [*T*, 0].

**Step 4 .**

The controls vector $\vec{u}$ is updated by entering the new state vector $\vec{x}$ and the costate values $\vec{\lambda }$ in the optimal characterisations.

**Step 5 .**

Convergence of the iterations is then checked by ascertaining whether successive iterations are negligibly close for some specified level of tolerance. Once this is achieved, then optimal control is said to have been achieved, the iteration is stopped, and the output values are recorded as solutions. Otherwise, the algorithm is set back to Step 2.

#### 4.1.1. Isolated Communities in Presence of Controls

**Figure 3:**
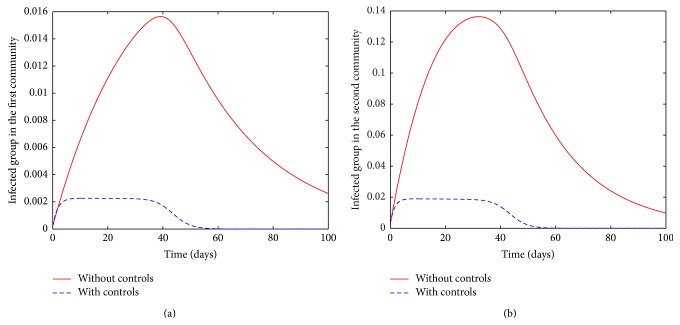
The infectious *I*_1_ (a) and *I*_2_ (b) in presence of controls (dashed line) and in absence of controls (solid line).

The communities are assumed to be isolated in this case if there is no movement or exchange of individuals between adjacent communities. However, we allow for recruitment from other areas and therefore the recruitment parameters are nonzero. In the simulations, we use the parameters in Table 1 and set all the parameters, describing movement to zero. The trajectories of the infected populations in both communities are given in Figure 3.

When all the controls are implemented, our results suggest that the time taken for the disease to be contained may be approximately half of the time required to contain the disease without controls, that is, by self-limitation. Although the infection is less devastating in the first community, it stays relatively longer in the community compared to the second community (cf. the solid lines in Figures 3(a) and 3(b)). It is plausible that, due to a high transmission in the second community, the disease depletes the susceptible pool at a much faster rate and in about 100 days, there are virtually no more susceptible people to infect for the chosen parameter values. In the model with controls however, the disease is contained at approximately the same time in both communities. Taking a closer look at dashed curves in Figures 3(a) and 3(b), one observes that the disease is contained slightly earlier in the first than the second community for the same level of effort. This scenario is a typical comparison of infections with high and low incidence.

The trajectories of the state space variables for the model with control, the adjoint, and the control values converge around relatively the same time. The iteration process terminates when these different variables converge sufficiently.

#### 4.1.2. Connected Communities in Presence of Controls

When the communities are connected, the parameters describing movement are all nonzero and there is exchange of individuals between the communities.

**Figure 4:**
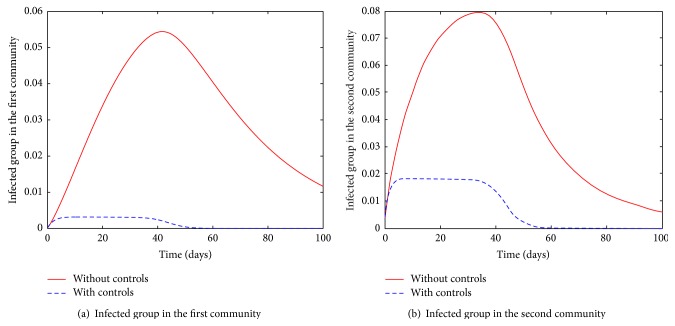
Infected groups in the two communities with and without controls.

Movement of susceptible and infected individuals is associated with not only an increase in severity but also a long duration of the infection in the two communities; see Figures 3 and 4. In presence of controls however, there is no observed significant difference in the disease severity and duration whether in isolated or connected community cases. For comparison, see dashed lines in Figures 3 and 4.

**Figure 5:**
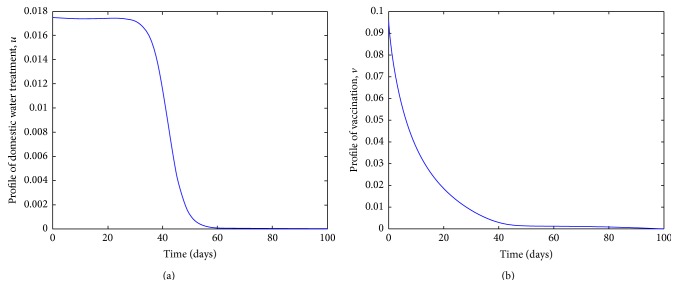
Profiles of the two most significant controls.

**Figure 6:**
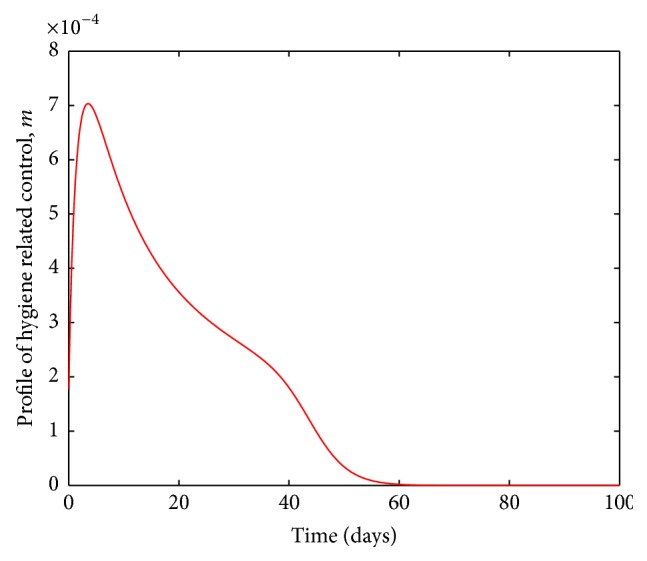
Profile of hygiene related control.

The profiles of the controls investigated in the model are indicated in Figures 5 and 6. The profiles indicate the need for more fundamental action at the beginning of the epidemic if the disease is to be contained. All the three indicated controls reduce the likelihood of susceptible individuals getting infected. The profiles for access to clean water and vaccination, Figures 5(a) and 5(b), respectively, indicate the importance of the two controls in containing the infection.

The profile for the control related to hygiene is deemed the least significant; see Figure 6. However, we reiterate that no effort is insignificant as long as it leads to reduction in the number of infections.

**Figure 7:**
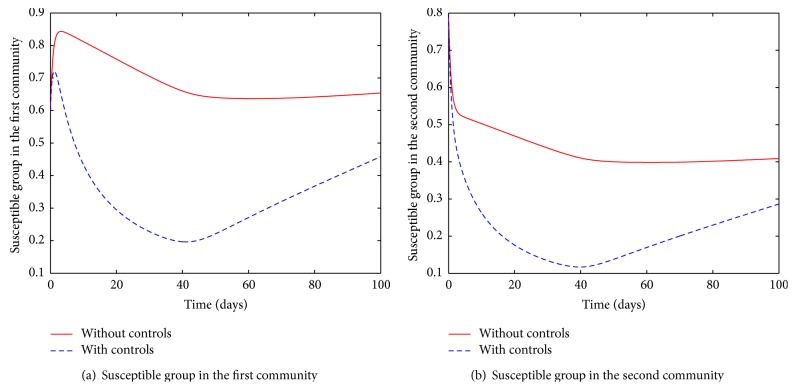
Susceptible populations in the two connected communities.

**Figure 8:**
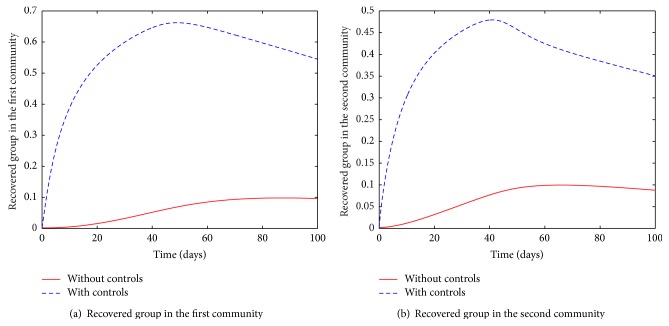
Recovered group in the two connected communities.

The susceptible groups in both communities are initially characterised by depletion followed by an increase, Figure 7. We note that the rapid depletion of the susceptible population is due to vaccination, which was assumed to be exponential in the model. As a result, the recovered group is replenished and reduces when the control is halted, Figure 8. By the end of the control period, the community is comprised of those susceptible and recovered. Once the infection is contained, it is assumed that the small group of people who could still be carrying the disease may pose a low threat of reintroducing it.

Although the general trajectories of the susceptible groups in the two communities are similar, there is a striking difference that ought to be highlighted. Even though there is an observed initial increase in the trajectory of the susceptible group in the first community, that of the second community only decreases. This trend is attributed to movement which is inclined towards the first community due to the better living conditions assumed, as well as the high incidence of the disease in the second community.

## 5. Conclusion

In this paper, a metapopulation model for cholera transmission characterised by exchange of individuals between communities is analysed. The community specific disease thresholds are given and their importance determining the existence and stability of equilibria of the model is highlighted. The conditions for annihilating the infection based on specific controls while keeping other parameters constant have been indicated. Our numerical results indicate that, in presence of controls, in case of an outbreak of the disease, the infection may be 8 times less devastating when compared to the case without controls. In addition, the duration of the infection in the community cannot be more than half the time it would in absence of controls. From the model results, we assert that implementation of preventive measures and treatment control protocols reduces the likelihood of recurrence of the infection in the communities. The recurrence of the infection is typical in the model without controls presented in [9], characterised by semiperiodic and synchronous fluctuations of the involved subpopulations.

The model presented in this paper is not short of shortfalls. We acknowledge the fact that controls across communities may not be uniform as assumed in the models. Controls efforts are often influenced by demographics of the community (including ageing which is associated with immune dysfunction) as well as the behaviour of individuals involved in the disease transmission dynamics. In addition, politics greatly affects the minority groups as well as social inequality which impacts the vulnerable groups, urbanization associated with overcrowding, inadequate infrastructure, and economic stochasticity among others. All these affect the bureaucracy associated with implementation of controls, putting up sustainable solutions resulting in heterogeneity. The model also assumes a negligible time difference between infection and becoming infectious as well as apparition of symptoms. Although such simplifying assumptions may be presumed feasible, their investigation is necessary to ascertain the effect on the epidemic size.

## Figures and Tables

**Table 1:** Nominal values of estimated parameter values used in the simulations.

Parameter	Range	Value	Units	Source
*π*_1_		*μ* × *N*_1_		N/A
*π*_2_		*μ* × *N*_2_		N/A
*β*_1_	0-1	0.00125		Assumed
*β*_2_	0-1	0.0125		Assumed
*K*	10^6^–10^9^	10^6^	Cells L^−1^	[10]
*μ*_1_, *μ*_2_		5.4 × 10^−5^	Day^−1^	[11, 12]
*δ*_1_	6.58 × 10^−4^–0.0182	0.0125	Day^−1^	[6, 13]
*δ*_2_	6.58 × 10^−4^–0.0182	0.045	Day^−1^	[6, 13]
*γ*_1_	0.031–0.059	0.045	Day^−1^	[6, 14]
*γ*_2_	0.031–0.059	0.035	Day^−1^	[6, 14]
*μ*_*p*_	1.017–1.083	1.06	Day^−1^	[10, 15–17]
*g*_1_, *g*_2_		0.73	Day^−1^	[10]
*α*_1_	0.031–0.059	0.045	Day^−1^	Assumed
*α*_2_	0.031–0.059	0.035	Day^−1^	Assumed
*σ*_1_, *σ*_2_	10–100	50.0	Cells mL^−1^ day^−1^ person^−1^	[10]

**Table 2:** Costs associated with permissible controls.

Coefficient	Cost value	Source
*ξ*_1_	R200 per percentage reduction in *I*_1_	Assumed
*ξ*_2_	R120 per percentage reduction in *I*_2_	Assumed
*X*	R80 per (level of water related treatment)^2^	[18]
*Y*	R50 per (vaccination rate)^2^	[19]
*Z*	R50 per (hand hygiene related infection reductions)^2^	[18]
